# Sea surface temperature and salinity from French research vessels, 2001–2013

**DOI:** 10.1038/sdata.2015.54

**Published:** 2015-10-13

**Authors:** Fabienne Gaillard, Denis Diverres, Stéphane Jacquin, Yves Gouriou, Jacques Grelet, Marc Le Menn, Joelle Tassel, Gilles Reverdin

**Affiliations:** 1 Ifremer, UMR 6523, LPO, CNRS/Ifremer/IRD/UBO, CS 10070, Plouzane F-29280, France; 2 US-Imago, Institut de Recherche pour le Developpement (IRD), BP 70, Plouzane 29280, France; 3 Service Hydrographique et Oceanographique de la Marine (SHOM), CS 92803, Brest Cedex 2 29228, France; 4 LOCEAN, Sorbone Universites, UPMC/CNRS/IRD/MNHN, 75005 Paris, France

**Keywords:** Climate change, Physical oceanography, Environmental sciences

## Abstract

French Research vessels have been collecting thermo-salinometer (TSG) data since 1999 to contribute to the Global Ocean Surface Underway Data (GOSUD) programme. The instruments are regularly calibrated and continuously monitored. Water samples are taken on a daily basis by the crew and later analysed in the laboratory. We present here the delayed mode processing of the 2001–2013 dataset and an overview of the resulting quality. Salinity measurement error was a few hundredths of a unit or less on the practical salinity scale (PSS), due to careful calibration and instrument maintenance, complemented with a rigorous adjustment on water samples. In a global comparison, these data show excellent agreement with an ARGO-based salinity gridded product. The Sea Surface Salinity and Temperature from French REsearch SHips (SSST-FRESH) dataset is very valuable for the ‘calibration and validation’ of the new satellite observations delivered by the Soil Moisture and Ocean Salinity (SMOS) and Aquarius missions.

## Background & Summary

Ocean salinity is a key parameter of the freshwater cycle of the earth system. It influences the ocean circulation by acting on the density field and is also used as a tracer of water mass origin^1^. Ocean salinity is one of the Essential Climate Variables (ECVs) identified by the Global Climate Observing System (GCOS). A global observing system based on profiling floats (ARGO)^2^ has been set up to monitor the water column temperature and salinity with typical resolution of 300 km in space and 10 days in time. The requirements for observing the near-surface are for higher time and space sampling because this layer interacts with the atmosphere, the ocean-land boundary and the ice, inducing fronts and small scale structures that can be very intense and rapidly changing^3–5^. The challenge of monitoring surface salinity at global scale has become a step closer with the launch of two satellites: SMOS (Europe) in November 2009 followed by Aquarius (US/Argentina)^6^ in June 2011. However, the in-situ salinity observing network must also increase the quantity, quality and timeliness of near surface measurements (upper five meters) to help calibrate and validate the satellite data and also to provide complementary data for regions where remotely sensed retrievals remain poor (e.g., coastal and high latitude areas).

Ships equipped with thermo-salinometers provide high resolution measurements along their track. These devices measure the conductivity and temperature of the water pumped in through the ship's seawater intake to deduce salinity. The nominal instrumental accuracy of the measurement is better than 0.01 PSS and the resolution close to 0.001 PSS, which is largely sufficient to capture the surface variability. However, siting of the instrument, stability of the electronics, sufficient flow through the conductivity cell, air bubbles and contamination by fouling and deposits may dramatically increase the measurement errors and even lead to discarding the data.

The Global Ocean Surface Underway Data programme (GOSUD) of the Joint Technical Commission for Oceanography and Marine Meteorology (JCOMM) and the International Oceanographic Data and Information Exchange (IODE) began in the early 2000’s with the main objective of developing the ocean surface measurements and assembling qualified datasets. The continuous data acquisition of thermo-salinometer data on board French Research Vessels was initiated in 1999. To ensure a data quality in accordance with current research standards, a methodology was developed, following what was done for the data collected on board merchant ships^7^ by the Sea Surface Salinity Observation Service (SO-SSS). The procedure described here has been designed for the research vessels by a group of French research institutes (Ifremer, IRD, INSU, SHOM).

To facilitate the maintenance of the equipment, a set of similar instruments has been selected: the SBE-21 device provides the salinity value and an additional temperature sensor is installed in the water inlet (generally a SBE-3S or SBE-38 sensor) to obtain the ocean temperature, that may differ from the temperature of the water when it reaches the conductivity cell inside the ship. The sensors are calibrated annually by the SHOM metrology service and returned to Seabird Electronics when non-conformities are detected, otherwise an offset and a slope are estimated and taken into account in the instrument software. Calibration uncertainties are calculated according to^8^ for salinity values.

On board ships operated by Ifremer/Genavir (at the present time all ships except the Beautemps-Beaupré), the installation and the operation of the instruments is performed by the crew. A similar system is applied on the ship operated by other partners. Instruments are cleaned at the beginning of each cruise and water samples are taken on a daily basis and later analysed in the SHOM laboratory. Reduced data are transmitted in real time to the Coriolis datacentre and full resolution data are archived on board and provided to the SISMER/Ifremer datacentre after the cruises. When the samples for a full year have been analysed, the delayed mode processing is performed. This includes a visual quality control of temperature and salinity, and the estimation of a correction to produce the corresponding ‘adjusted’ variable. At the end of the processing the dataset is uploaded into the GOSUD database.

## Methods

We present here the delayed mode processing performed on the dataset collected by the Research Vessels presently in the project (see Table 1). All data available up to October 2014 have been taken into account and we plan to update the database annually.

**Figure 1:**
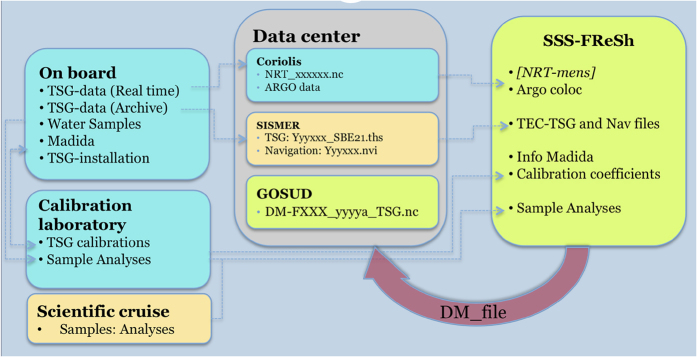
Thermo-salinometer delayed mode processing workflow. On French research vessels, data acquisition relies on the ship operator and the calibration laboratory. The data centre collects real time data and full resolution archives. The delayed mode processing team assembles the necessary information to provide qualified and adjusted data sets to the GOSUD database.

The data processing follows the method developed at SO-SSS (http://www.legos.obs-mip.fr/observations/sss) and relies on the software package TSG-QC which is freely available (http://www.ird.fr/us191/spip.php?article63). Further developments have been included over time and the processed datasets were used for studies in the Pacific Ocean^9,10^ and in the Atlantic^11,12^. We describe here the specific choices made to process the Research Vessels dataset. The full processing cycle is sketched in Fig. 1.

### Preparing the GOSUD delayed mode files

For data prior to 2012, the monthly ‘Near Real Time’ data files were downloaded from the Coriolis database. These data have been under sampled on board using a median filter (in most cases the period is 5 min) and converted to GOSUD NetCdf format by Coriolis (‘NRT_’ files in Fig. 1). Since 2012, we use the full resolution (6 s) thermo-salinometer data archived on board and the delayed mode processed navigation, that we download (‘.ths’ and ‘.nvi’ files in Fig. 1) from the SISMER cruises database. We perform the under sampling with a median filter over 90 s and check that the data quality is sufficient. If not, the sampling period would be increased, which has not been necessary with the current dataset. We then convert the dataset to the GOSUD NetCdf format.

The files downloaded from the data centre contain data assembled by month (‘NRT_’ files) or by cruises (‘.ths’ and ‘.nvi’ files). For the processing, all data corresponding to the same year and the same instrument (identified by its serial number) are assembled in a single file. The operator of the French Research vessels (Genavir) maintains a database (Madida) that holds information on the instrument servicing such as installation dates, calibration coefficients, offset and slopes of the instruments, dates of cleaning as well as any event of importance for the data quality. Before assembling the annual files, we extract from Madida the information relevant to the dataset.

### Quality control

The variables measured with the TSG system are summarised in Table 2 (available online only).

- Sea Surface Temperature (SSTP) is the temperature of the seawater at the water intake. When the water is pumped inside the ship, it represents the temperature of the ocean in the vicinity of the water intake, taking into account that the ship displacement produces a mixing of the water mass. When the seacock is closed, or the pump stopped, it gives the temperature of the water volume trapped close to the temperature sensor.
- Sea Surface Jacket Temperature (SSJT) is the temperature of the water volume inside the TSG. SSJT is used to deduce salinity from the conductivity measurement. It may differ from SSTP because of heat exchange on the way to the TSG and in the TSG room. The temperature change on the way depends on the flow rate, on the volume of water in the circuit and on the temperature difference between the seawater and the ambient temperature.
- Sea Surface Practical Salinity (SSPS) is the salinity of the water inside the TSG deduced from the conductivity measurement, and SSJT. When the flow rate is sufficient, SSPS represents the ocean salinity in the vicinity of the water intake, otherwise it is the salinity of the water volume trapped in the TSG.

**Figure 2:**
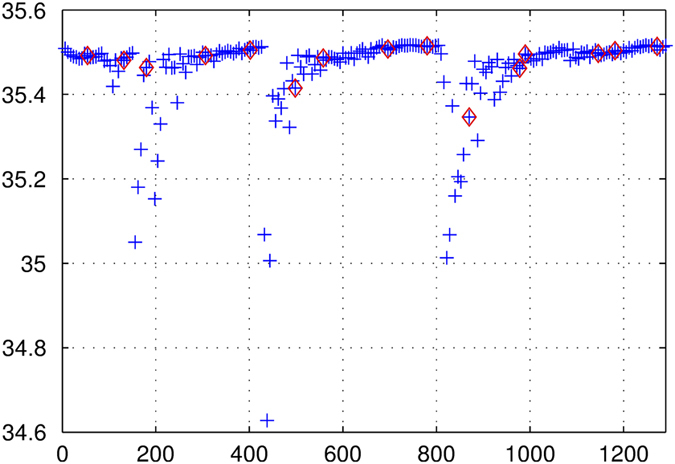
Effect of bubbles on the salinity reported by the TSG. When the ship is heading into strong waves, air bubbles on the conductivity cell decrease the true salinity value. We show here the full resolution salinity data (in blue) and the reduced data (in red), in PSS as a function of time, given in seconds.

The quality control is performed using the ‘QC flag menu’ of TSG-QC software that allows quality flags to be applied to the variables (See Table 3 (available online only) for the QC definition). Several cases are considered:

1. **Problems on the water flow**: We first eliminate the periods during which the flow appears to be insufficient. **The corresponding SSPS QC-flag values are set to 4 (bad data)**. These events are characterized by a large and increasing difference between SSTP and SSJT, and values of SSPS that remain nearly constant. The standard criterion used for the SSTP-SSJT difference is 0.2 °C, but it may vary with the ship and the area. In rare cases, the flow was detected as slightly insufficient and the QC-flag value was set to 3. We are currently installing flowmeters on the ships to obtain a direct measure of the flow rate and define an objective criterion based on a threshold value.
2. **Air bubbles**: During severe sea state conditions, air bubbles reach the conductivity cell, inducing a drop of the measured conductivity. The SSPS reported by the TSG decreases accordingly, to a slightly underestimated value (of the order of 0.1) when only small bubbles are present or to several PSS, in the case of stronger waves. It can even reach 0 in extreme conditions. The normal SSPS value is recovered within a few minutes, the frequency of these events depends on the sea state. In that case, **SSPS QC-flag values are set to 2, 3 or 4**, depending on the magnitude of the salinity decrease. The effect of air bubbles on the full resolution data is illustrated in Fig. 2.
3. **Shells in the conductivity cell**: It may happen that shells or other solid matter get trapped inside the conductivity cell, inducing erroneous conductivity measurement with salinity drops by several tenths of PSS or more, a step-like recovery occurs as the debris change position within the conductivity cell or are flushed out. In that case, **SSPS QC-flag values are set to 4.**
4. **Harbour**: Measurements taken in the harbour or estuaries are difficult to qualify because of the high variability due to tides and river run offs, the corresponding **SSTP and SSPS QC-flag values are set to 6.**

### Including external measurements

The water samples collected by the crew are stored using double cap bottles distributed by Ocean Scientific International Ltd. (OSIL). They are analysed in the SHOM laboratory 3 to 6 months later. In some cases, scientific groups contribute by providing external measurements, either independent water samples analysed on board with their salinometer (AutoSal or Portasal) or near surface salinity obtained with a CTD (conductivity-temperature-depth profiling instrument of accuracy higher than the TSG). These datasets based on measurements performed on the ship, but independent from the TSG, are included in the NetCdf file as ‘external measurements’ and will be considered as a reference on which we will adjust the TSG data.

Prior to the adjustment, a visual quality control is applied to the external dataset. In some rare instances water samples diverge from the records by more than 5 standard deviations, in which case they are considered invalid and flagged as ‘Bad’ (QC=4). These water sample values are very different from what is measured by the TSG. The first explanation is a modification of the salinity of the water sample. With the OSIL double cap, evaporation is no longer a problem but the sampling or analysis conditions may have introduced salt crystals or fresh water. However, the most common error is due to a bad labelling of the sample (wrong date or time). Double checks are now performed on board to reduce this type of error. We also find some samples, which are very likely accurate, but diverge by 3 to 4 standard deviations from the value reported by the TSG. It happens often when salinity changes rapidly over the time of collection of the sample, introducing a large uncertainty. These are marked as ‘probably bad’ (QC=3), and are not used to estimate the linear fit. Depending on the year and vessel, 0 to 10% of the water samples were excluded from the fit but the number of excluded samples is decreasing with time and experience of the crew members.

The Argo program maintains a network of more than 3000 profiling floats (3700 currently). The time sampling of Argo floats is 10 days and the average float to float distance is 300 km, with the last pumped measurement made between 8 and 5 metres. The data quality of Argo measurements is high because the sensor reports temperature and salinity to approximately 0.01 °C and 0.01 respectively, with little effect of fouling since the float spends most of its time at great depth (1000 m). We have thus considered using Argo data as an additional ‘external’ measurement. To do so, we have extracted the Argo measurements from floats found within 5 days and closer than 50 km to the TSG measurements. However, because of large time and space variability of sea-surface properties (fronts, mixing from the winds, rain) the data can rarely be used to estimate biases in the TSG instrumental record unless the float is very close in time and space to the ship.

**Figure 3:**
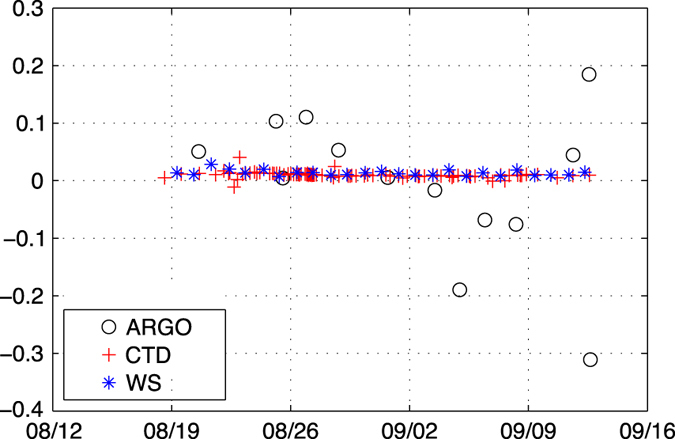
Difference between the salinity reported by the TSG and the external measurements. During the Strasse cruise on board the Thalassa in 2012 a large number of CTD data were collected (in red), they are in excellent agreement with the daily water samples (in blue) collected by the ship crew and reveal a 0.01 offset of the TSG, with very low standard deviation. Argo measurements show a strong dispersion.

During the Strasse cruise^13^, on board the Thalassa in the North Atlantic maximum salinity area during August-September 2012, the water samples taken by the crew were complemented by numerous CTD data provided by the scientific team. Moreover, the density of Argo floats during that cruise was high (some floats were launched from the ship). The differences ΔS between the external measurements and the TSG measurements shown in Fig. 3 reveal an excellent agreement between the samples (bottles) and the CTD, converging to an estimate of 0.01 offset of the TSG (and no drift), and a standard deviation of 0.005 (Table 4 (available online only)). The Argo measurements show a much larger scatter (standard deviation of 0.13), because they did not sample the same water mass.

### Adjustment on water samples

The systematic difference that appears between the salinity values reported by the thermosalinometer and the external measurements (water samples or CTD) can be due to instrumental drift (usually small) and more commonly to biofouling.

On the research vessels, the thermosalinometer is carefully maintained. The tank and conductivity cell are cleaned at the beginning of each cruise and sometimes during the cruise. The thermosalinometer is stopped before entering the harbour to preserve it from pollution. Then although some fouling develops over the duration of the cruise, it remains limited and never reaches exponential growth. The basic time period for estimating the drift is thus the period separating two cleaning operations, which is between a week and a month. On the many datasets we have processed, the drift observed was nearly linear (and even constant in many cases) as seen during the Strasse cruise (Fig. 3). In cases where we noted continuity in the drift between two consecutive cruises, they were processed as a single period, leading to a more stable estimate of the drift since more samples could be used.

We chose not to use Argo measurements in the adjustment procedure, for that reason they were flagged as ‘probably bad’ before running TSG-QC adjustment. They are used only as a visual check. Only the TSG data flagged ‘good’, ‘probably good’ or ‘harbour’ are adjusted. Two types of correction may be applied. When the difference between the TSG measurement and the samples appears stable over the period considered, a constant offset is proposed (the mean of the differences) otherwise, a linear drift is estimated. An error based on the scatter of the differences with water samples is attached to the variable. In cases for which no samples are available and the time series appear consistent with ARGO profiles, a 0 offset and a 0.05 to 0.1 error, corresponding to the typical drift over a cruise for the ship, is assumed. This allows us to provide a complete time series of the ‘adjusted variable’.

**Figure 4:**
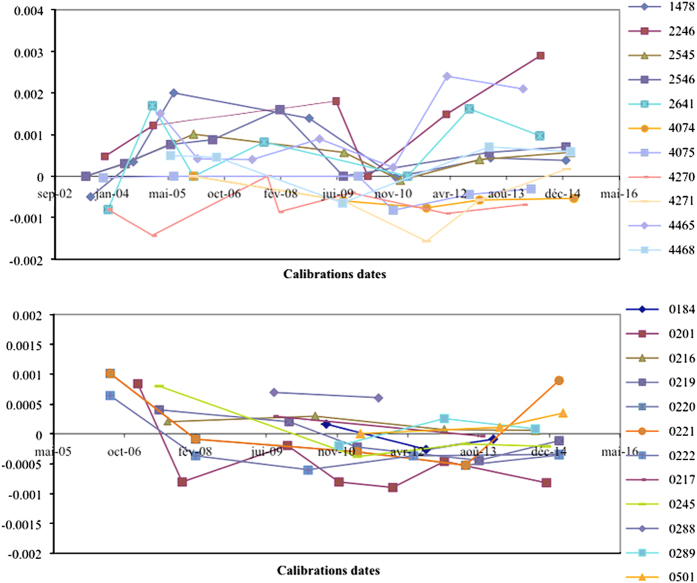
Difference between the TSG temperature and the laboratory reference temperature. The temperature sensors (SBE-38 and SBE-3S) used on the research vessels are regularly controlled in the SHOM metrology service. The sensor drift observed on all instruments from 2002 to 2014 is within the specifications declared by the manufacturer (0.001 °C in 6 months).

The inlet temperature adjusted value is identical to the ‘raw’ value, considering that stability of the temperature sensors declared by the manufacturer is 0.001 °C in 6 months (see http://www.seabird.com/sbe38-thermometer). This stability has been verified on all the SBE-3S and SBE-38 devices followed in the calibration laboratory (Fig. 4).

## Data Records

The data are recorded in the GOSUD NetCdf format described on the GOSUD web page: http://www.gosud.org/Documents/Format-and-templates.

For each vessel, and each year, one file is created per installation of thermosalinometer. The naming convention is as follows:

DM_**nnnn** _**yyyya**_TSG.nc: where **nnnn** is the ship call sign, **yyyy** the year and the additional letter indicate the file order and can be **a**, b, c.

The meta-data appear in the NetCDF global attributes, and the main variables are described in Table 5 (available online only). The SSST-Fresh 2014 dataset is available to the public through an unrestricted repository at the PoleOcean Portal (Data Citation 1).

## Technical Validation

This worldwide sea surface salinity dataset covering more than a decade can be systematically compared to equivalent measurements performed by independent devices. As explained in the previous section, the point to point comparison with Argo data direct measurements is a possibility, subject to the limitation of having only a small number of co-localized observations and the question of sampling a different water mass. We chose instead to compare our SSS-Fresh dataset to a gridded product of salinity based on in-situ observations but that did not include TSG: the ISAS-13 monthly analysis^14^ (Data Citation 2). The *in situ* observations are mostly from Argo floats, but some mooring data, CTDs and marine mammals T-S profiles were used. The salinity field at 10 meters depth was selected for being the best sampled near-surface level. The horizontal grid resolution is 0.5°, but the analysis process includes a horizontal smoothing with an e-folding scale of 300 km in the N-S direction and 600 km in the E-W direction in the tropical band, that decreases poleward (it is set to 6 times the Rossby Radius). We are thus comparing a local measurement to a large scale estimate of the salinity field and the differences between the 2 quantities include the scales that the gridded product has filtered out.

**Figure 5:**
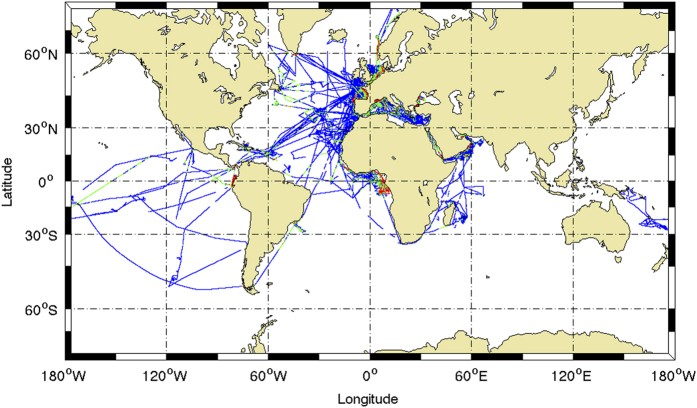
Difference between the TSG salinity measurements and the salinity gridded field from ISAS-13 monthly analysis. ΔS is defined as the salinity difference with ISAS-13, computed at each TSG data position. In blue |ΔS|≤0.5, in green 0.5<|ΔS|≤2, in red |ΔS|>2. |ΔS| is expressed in PSS. The period considered is defined by ISAS-13 availability and runs from 2002 to 2012.

**Figure 6:**
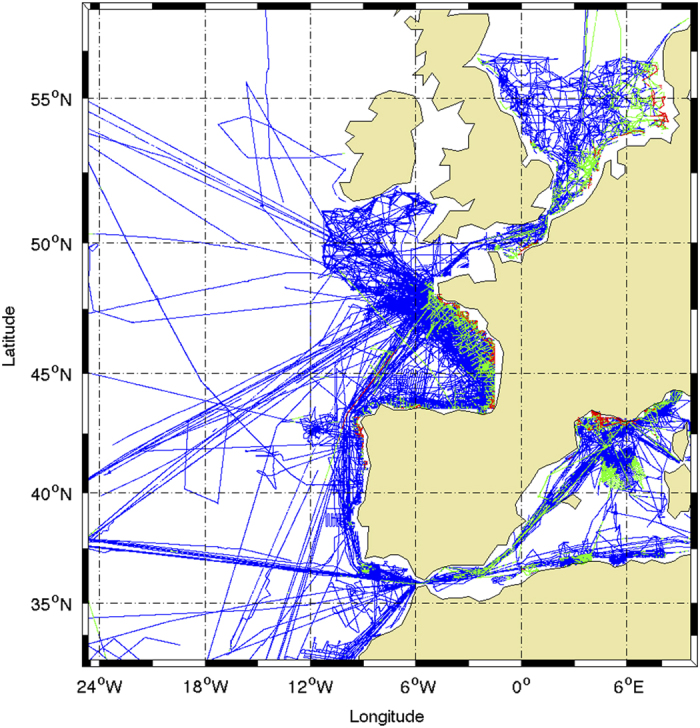
Difference between the TSG salinity and ISAS-13 monthly analysis in coastal areas. This figure is a zoom of Fig. 4 in the North-East Atlantic showing that the higher values of salinity anomalies relative to ISAS-13 are found in coastal areas. Note also the dense sampling in the North Sea, Bay of Biscay and Gulf of Lion.

The SSST-Fresh to ISAS13 differences have been sorted in 3 main classes: very high values (more than 2 PSS), large values (0.5 to 2 PSS) and moderate values (less than.5 PSS). The distribution of these three classes is mapped (Fig. 5) for all ships during the period 2002–2012. The very high values (1 to 5% of the data depending on the ship) are observed in the most coastal areas (Fig. 6) and in the Gulf of Guinea where river discharges or ice melt produce strong and localized freshening, not captured by the large scale Argo dataset. The large values (9 to 14% of the data depending on the ship) are seen in the same regions but over more extended areas. They also occur in the tropical band where they might be explained by the precipitations not captured by the Argo network time and space sampling.

**Figure 7:**
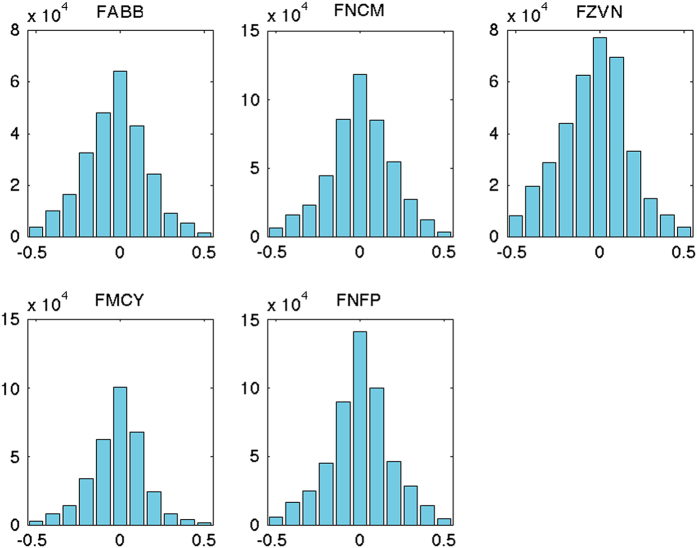
Distribution of TSG-ISAS-13 differences for each ship. The distribution is shown only for the values |ΔS|≤0.5.

Finally, for the moderate values (ΔS<0.5 PSS) the distribution by ship is analysed, in order to detect a systematic bias (Table 6 (available online only) and Fig. 7). The mean anomalies are very low (less than 0.03 PSS) and often slightly negative (TSG value is lower than ISAS-13 value), that can be explained by the vertical stratification and a difference between the near surface value (3–5 m) captured by the TSG and the lower ARGO estimate (10 m). The dataset appears globally of good quality, with no detectable biases. The standard deviation in the difference is between 0.16 and 0.20 PSS and corresponds to the meso-scale variability. Skewness is slightly negative, indicating a lack of symmetry with more negative than positive values. The excess kurtosis is low, showing that the shape of the distribution is close to normal except for ‘Le Pourquoi-Pas’ (FMCY), where it is more ‘peaked’.

## Usage Notes

The user should read data quality flags and errors. We also suggest using the ‘adjusted’ variables.

## Additional Information

Tables 2–6 are only available in the online version of this paper.

**How to cite this article:** Gaillard, F. *et al.* Sea surface temperature and salinity from French research vessels, 2001–2013. *Sci. Data* 2:150054 doi: 10.1038/sdata.2015.54 (2015).

## Supplementary Material



## Figures and Tables

**Table 1:** List of research vessels considered in the project and brief description of the equipment.

**Ship name/Call sign**	**Period processed**	**Salinity sensor type**	**Depth of S intake**	**Temperature sensor type**	**Depth of T sensor**
Pourquoi-Pas?FMCY	2006–2013	SBE-21	5.3	SBE-38	5.3
L’AtalanteFNCM	2003–2013 (no samples before 2003)	SBE-21	5.0	SBE-38	5.0
Beautemps-BeaupréFABB	2004–2012 (2013 samples not available in oct 2014)	SBE-21	2.5	SBE-3S	2.5
ThalassaFNFP	2001–2013	SBE-21	4.5	SBE-3S	4.5
Le SuroîtFZVN	2001–2013 (no data in 2010)	SBE-21	2.2	SBE-3S	2.2

**Table 2:** Variables measured with the thermo-salinometers (in bold), in order to approach the Ocean Sea Surface Temperature (SST) and Salinity (SSS)

**Temperature variable**	**Salinity variable**	**Location**	**Flow**
*SST*	*SSS*	Ocean	↓↓
**SSTP**		Ship Intake	
**SSJT**	**SSPS**	TSG, located in TSG Room	

**Table 3:** Definition of the quality control flags

0	No QC was performed
1	Good data
2	Probably good data
3	Probably bad data
4	Bad data
5	Value changed
6	Harbour
7	Not used
8	Interpolated value
9	Missing value

**Table 4:** Mean and standard deviation of the difference between the thermosalinometer salinity (SSPS) and the various types of external salinity

	**Mean ΔS**	**RMS ΔS**	**Number of data**
Argo	−0.008	0.131	13
CTD	+0.010	0.005	87
Samples	+0.013	0.005	25

**Table 5:** Name and description of the main variables included in the NetCdf files

**Variable name**	**Unit**	**Description**
DATE	‘yyyymmddHHMMSS’	Date/time of TSG measurement
LATX	Decimal degree	Latitude of TSG measurement
LONX	Decimal degree	Longitude of TSG measurement
SSPS	PSS	TSG salinity measurement
SSPS_QC	See Table 3	TSG salinity measurement QC
SSPS_ADJUSTED	PSS	Adjusted TSG salinity
SSPS_ADJUSTED_ERROR	PSS	Error on Adjusted TSG salinity
SSTP	Celsius	Intake temperature measurement
SSTP_QC	See Table 3	Intake temperature measurement QC
SSTP_ADJUSTED	Celsius	Adjusted intake temperature
SSTP_ADJUSTED_ERROR	Celsius	Error on Adjusted intake temperature

**Table 6:** Statistics by ship

	**% ΔS<=0.5**	**% 0.5<ΔS<=2**	**% ΔS>2**	**ΔS mean PSS**	**ΔS Std PSS**	**Skewness**	**Excess Kurtosis**
FABB	84.5	12.1	3.4	−0.029	0.18	−0.06	0.05
FNCM	84.3	10.9	4.8	0.001	0.19	−0.12	0.08
FZVN	81.6	14.0	4.4	−0.033	0.20	−0.05	−0.17
FMCY	89.0	9.2	1.8	−0.016	0.16	−0.17	0.80
FNFP	89.7	9.2	1.1	0.002	0.18	−0.08	0.27
Column 1–3, percentage of salinity anomaly (TSG-ISAS-13 differences) in each of the three classes. Column 4–7: characteristics of the distribution within class 1 (ΔS<=0.5).							
